# Cardioprotection Conferred by Sitagliptin Is Associated with Reduced Cardiac Angiotensin II/Angiotensin-(1-7) Balance in Experimental Chronic Kidney Disease

**DOI:** 10.3390/ijms20081940

**Published:** 2019-04-20

**Authors:** Juliana Isa Beraldo, Acaris Benetti, Flávio Araújo Borges-Júnior, Daniel F. Arruda-Junior, Flavia Letícia Martins, Leonardo Jensen, Rafael Dariolli, Maria Heloisa Shimizu, Antonio C. Seguro, Weverton M. Luchi, Adriana C. C. Girardi

**Affiliations:** 1 Heart Institute (InCor), University of São Paulo Medical School, São Paulo 05403-900, SP, Brazil; juliana_isab@hotmail.com (J.I.B.); acaris.ben@gmail.com (A.B.); flavio44jr@gmail.com (F.A.B.-J.); danielarruda.junior@gmail.com (D.F.A.-J.); flaviabiomack@gmail.com (F.L.M.); jensenleonardo@gmail.com (L.J.); rdariolli@gmail.com (R.D.); wmluchi@hotmail.com (W.M.L.); 2 Department of Nephrology (LIM 12), University of São Paulo Medical School, São Paulo 05403-900, SP, Brazil; hmshimizu@gmail.com (M.H.S.); trulu@usp.br (A.C.S.); 3 Department of Internal Medicine, Federal University of Espírito Santo (UFES), Vitoria 29040-090, Espírito Santo, Brazil

**Keywords:** dipeptidyl peptidase IV, 5/6 renal ablation, renin-angiotensin system, cardiorenal syndromes

## Abstract

Dipeptidyl peptidase IV (DPPIV) inhibitors are antidiabetic agents that exert renoprotective actions independently of glucose lowering. Cardiac dysfunction is one of the main outcomes of chronic kidney disease (CKD); however, the effects of DPPIV inhibition on cardiac impairment during CKD progression remain elusive. This study investigated whether DPPIV inhibition mitigates cardiac dysfunction and remodeling in rats with a 5/6 renal ablation and evaluated if these effects are associated with changes in the cardiac renin-angiotensin system (RAS). To this end, male Wistar rats underwent a 5/6 nephrectomy (Nx) or sham operation, followed by an 8-week treatment period with the DPPIV inhibitor sitagliptin (IDPPIV) or vehicle. Nx rats had lower glomerular filtration rate, overt albuminuria and higher blood pressure compared to sham rats, whereas CKD progression was attenuated in Nx + IDPPIV rats. Additionally, Nx rats exhibited cardiac hypertrophy and fibrosis, which were associated with higher cardiac DPPIV activity and expression. The sitagliptin treatment prevented cardiac fibrosis and mitigated cardiac hypertrophy. The isovolumic relaxation time (IRVT) was higher in Nx than in sham rats, which was suggestive of CKD-associated-diastolic dysfunction. Sitagliptin significantly attenuated the increase in IRVT. Levels of angiotensin II (Ang II) in the heart tissue from Nx rats were higher while those of angiotensin-(1-7) Ang-(1-7) were lower than that in sham rats. This cardiac hormonal imbalance was completely prevented by sitagliptin. Collectively, these results suggest that DPPIV inhibition may delay the onset of cardiovascular impairment in CKD. Furthermore, these findings strengthen the hypothesis that a crosstalk between DPPIV and the renin-angiotensin system plays a role in the pathophysiology of cardiorenal syndromes.

## 1. Introduction

Chronic kidney disease (CKD) and its associated morbidity is a global health burden. Cardiovascular disease (CVD) is the most common cause of death among the CKD population. A decline in the glomerular filtration rate, an increase in the urinary albumin-to-creatinine ratio and an increase in blood pressure are independently associated with an increased risk of cardiovascular morbidity and mortality and thus remain critical targets of study for the development of novel therapies.

Diabetic nephropathy is the most common cause of CKD. Dipeptidyl peptidase IV (DPPIV) inhibitors, also called gliptins, are widely used for type 2 diabetes mellitus (T2DM) management. These antidiabetic agents have been shown to be safe and effective to treat T2DM patients with common renal and cardiovascular risk factors. Moreover, the Saxagliptin Assessment of Vascular Outcomes Recorded in Patients with Diabetes Mellitus–Thrombolysis in Myocardial Infarction 53 (SAVOR-TIMI 53) trial [1,2] showed that DPPIV inhibition reduces albuminuria in T2DM subjects with CKD, suggesting that the DPPIV inhibitors may confer renoprotective effects that extend beyond glycemic control. Indeed, treatment with the DPPIV inhibitors ameliorates renal dysfunction and structural damage in animal models of CKD that do not display hyperglycemia [3,4,5].

DPPIV inhibition also appears to reduce cardiac fibrosis in CKD rats [6] and to improve renal function in experimental models of heart failure [7,8,9], implicating DPPIV activity in the complex interplay between the heart and kidney in the setting of cardiorenal syndromes. However, the mechanisms underlying the role of this peptidase in the pathophysiology of cardiac and renal failure have not yet been elucidated. DPPIV decreases the bioavailability of peptides with cardiorenal functions, including glucagon-like peptide-1 (GLP-1), brain natriuretic peptide (BNP) and stromal cell-derived factor-1 (SDF-α) [9,10,11,12,13]. Several independent lines of evidence have demonstrated that a reduction in the biological activity of these DPPIV substrates may be associated with a progressive decline in the glomerular and proximal tubule function, increasing albuminuria and cardiac dysfunction.

The renin-angiotensin system (RAS) is a key mediator of both CKD and CVD progression [14,15,16]. Recent reports show that angiotensin II (Ang II) stimulates renal DPPIV activity both in vitro and in vivo via the angiotensin II type 1 (AT1) receptor [17] and that DPPIV inhibition can reduce the levels of circulating Ang II in spontaneously hypertensive rats [18]. These studies therefore raise the intriguing possibility of a crosstalk between the DPPIV and RAS activation in cardiorenal pathologies. In light of the above findings, the present study tested the hypothesis that DPPIV inhibition attenuates cardiac dysfunction in rats with a 5/6 renal ablation, an experimental model of CKD that is not associated with hyperglycemia. In addition, we investigated whether the cardioprotective effects of DPPIV inhibition are associated with changes in relevant components of the RAS system in the heart of CKD rats. 

## 2. Results

### 2.1. DPPIV Inhibition Attenuates Renal Dysfunction and the Increase in Blood Pressure in CKD Rats

As shown in Table 1, body weight gain did not change significantly among the four groups of rats over the 8-week study. A significant elevation of serum creatinine and urea levels and a reduction in the GFR were observed in Nx rats compared to sham rats. Additionally, Nx rats exhibited much greater proteinuria and albuminuria in comparison to sham animals. The administration of sitagliptin to Nx rats (Nx + IDPPIV) induced remarkable renoprotective effects, which included attenuating the increase in serum creatinine and urea, the decrease in the GFR and the increase in proteinuria and albuminuria that were observed in Nx rats. The results in Table 1 also show that sitagliptin exerted an anti-hypertensive effect by attenuating the blood pressure increase in CKD rats. On the other hand, no differences in the renal function or blood pressure have been observed between sham rats treated with vehicle or with sitagliptin. Fasting blood glucose did not differ among the four groups of rats, indicating that the cardiorenal effects of sitagliptin were not related to changes in glycemia.

### 2.2. Cardiac DPPIV Activity and Expression Are Upregulated in CKD Rats

**Figure 1:**
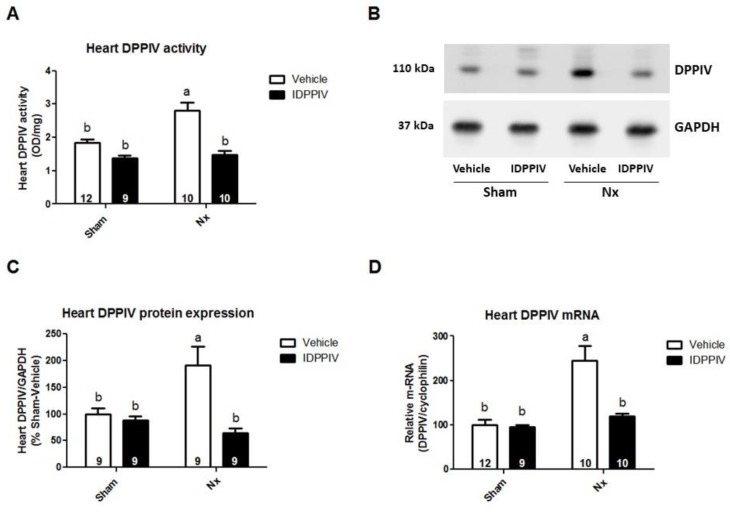
Cardiac dipeptidyl peptidase IV (DPPIV) activity and expression are upregulated in chronic kidney disease (CKD) rats. (**A**) DPPIV activity was measured using colorimetry in the heart tissue from sham and CKD rats (Nx) treated with vehicle or sitagliptin (IDPPIV); (**B**,**C**) the DPPIV protein level in the heart tissue from sham, sham + IDPPIV, Nx and Nx + IDPPIV rats was evaluated by immunoblotting; (**B**) representative immunoblot; (**C**) graphical representation of the relative levels of DPPIV protein in the rat. The GAPDH protein level was used as an internal control for normalization*. n* = 6 rats/group; (**D**) graphical representation of the relative gene expression of DPPIV in the heart tissue from sham, sham + IDPPIV, Nx and Nx + IDPPIV rats. The levels of mRNA of DPPIV were measured using quantitative PCR, and cyclophilin was used as an internal control. The number of rats per experimental group is indicated in the bars. The data represent the mean ± SEM. Bars with different lowercase letters are significantly different (*p* < 0.05).

The serum DPPIV activity did not differ between Nx and sham rats (Table 1). As expected, the DPPIV activity was inhibited by the sitagliptin treatment. Interestingly, the cardiac DPPIV activity (Figure 1A), protein abundance (Figure 1B,C) and mRNA expression (Figure 1D) were significantly increased in Nx rats compared to sham rats. In sitagliptin-treated Nx rats, the cardiac DPPIV activity, protein and mRNA expression were lower than in Nx rats and similar to that in sham rats.

### 2.3. DPPIV Inhibition Mitigates Cardiac Remodeling and Diastolic Dysfunction in CKD Rats

**Figure 2:**
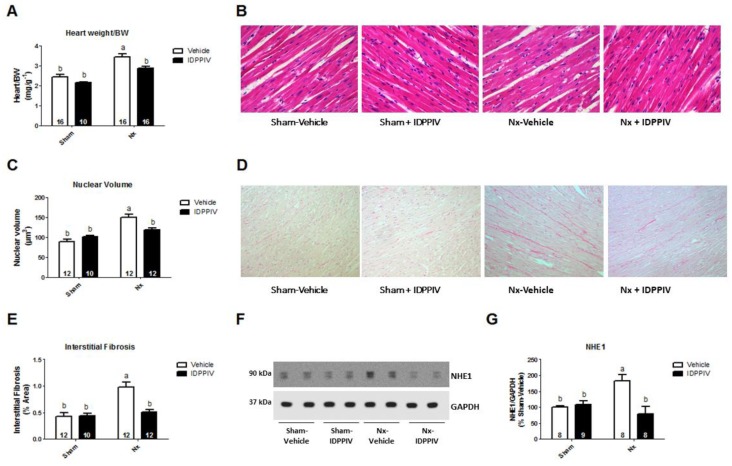
Treatment with sitagliptin attenuates cardiac remodeling in CKD rats. (**A**) The ratio between the heart weight (mg) and body weight (g) was determined in sham and CKD rats treated with vehicle or sitagliptin; (**B**,**C**) cardiac hypertrophy was assessed in sham, sham + IDPPIV, Nx and Nx + IDPPIV rats by measuring the cardiomyocyte nuclear volumes in heart sections stained with hematoxylin-eosin (400× magnification); (**D**,**E**) cardiac interstitial fibrosis was evaluated in heart sections stained with Picrosirius red (200× magnification); (**F**,**G**) the Na^+^/H^+^ exchanger isoform 1 (NHE1) protein level in the heart tissue from sham, sham + IDPPIV, Nx and Nx + IDPPIV rats was evaluated by immunoblotting; (**F**) representative immunoblot; (**G**) graphical representation of the relative levels of NHE1 protein in the rat. The GAPDH protein level was used as an internal control for normalization. The number of rats per experimental group is indicated in the bars. The data represent the mean ± SEM. Bars with different lowercase letters are significantly different (*p* < 0.05).

Figure 2 shows the measurements of cardiac mass, histological analysis of the myocardium and immunoblotting assays to evaluate the expression of the Na^+^/H^+^ exchanger isoform 1 (NHE1), which has been associated with maladaptive heart hypertrophy [19]. The heart weight as a function of body weight (HW/BW) was significantly increased in Nx rats compared to sham rats. In sitagliptin-treated 5/6 renal ablated-rats, the HW/BW was lower than that in Nx rats (2.87 ± 0.08 vs. 3.45 ± 0.14 mg/g, *p* < 0.05). In addition, there were no statistically significant differences in the HW/BW ratio between sitagliptin-treated 5/6 renal ablated-rats and sham rats (Figure 2A).

A histological analysis of the cardiac tissue was performed to evaluate the effect of DPPIV inhibition on cardiac hypertrophy and interstitial fibrosis of 5/6 renal ablated rats (Figure 2B–E). As shown in Figure 2B,C, the average cardiomyocyte nuclear volume in Nx rats was significantly increased compared to that in sham-operated rats. The increase in the cardiomyocyte nuclear volume induced by renal ablation was attenuated by sitagliptin. Furthermore, Nx rats had a higher percentage of interstitial collagen in the myocardium than sham rats, and this increase was completely prevented by sitagliptin (Figure 2D,E). As illustrated in Figure 2F,G, the cardiac NHE1 protein abundance was higher in Nx rats than in vehicle-treated sham rats (183 ± 18 vs. 100 ± 5%, *p* < 0.05) and sitagliptin-treated sham rats (183 ± 18 vs. 108 ± 11%, *p* < 0.05), whereas sitagliptin completely prevented the cardiac NHE1 upregulation in rats subjected to renal ablation (183 ± 18 vs. 79 ± 23%, *p* < 0.05).

**Figure 3:**
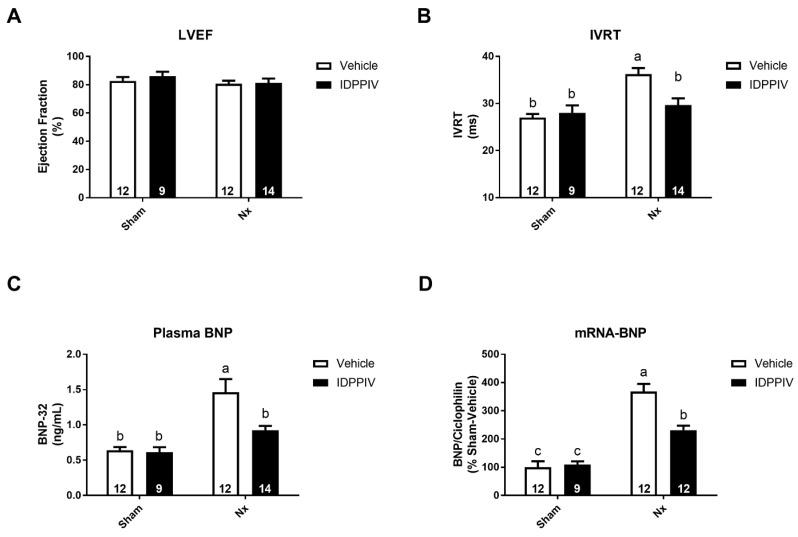
The effect of sitagliptin on the cardiac function and B-type natriuretic peptide (BNP) levels in CKD rats. (**A**,**B**) Doppler echocardiography was performed in sham and CKD rats treated with vehicle or sitagliptin; (**A**) left ventricle (LV) ejection fraction, as a marker of LV systolic function; (**B**) isovolumic relaxation time (IVRT), as a marker of LV diastolic function; (**C**,**D**) the levels of systemic and cardiac BNP were assessed in the four groups of rats; (**C**) serum BNP levels were determined by ELISA in sham, sham + IDPPIV, Nx and Nx + IDPPIV rats; (**D**) graphical representation of the relative gene expression of BNP in the heart tissue from sham, sham + IDPPIV, Nx and Nx + IDPPIV rats. The levels of BNP mRNA were measured using quantitative PCR, and cyclophilin was used as an internal control. The number of rats per experimental group is indicated in the bars. The data represent the mean ± SEM. Bars with different lowercase letters are significantly different (*p* < 0.05).

To evaluate whether the effects of sitagliptin on the cardiac structure of renal ablated rats were accompanied by functional changes, the cardiac performance was assessed in this setting using the echocardiography analysis (Figure 3). At the end of the 8-week treatment period, both groups of nephrectomized rats displayed a similar left ventricular ejection fraction (Figure 3A) as the sham group, providing evidence for the sustained systolic function. The isovolumic relaxation time (IVRT) (Figure 3B) was determined as a marker of the diastolic function of the left ventricle (LV). The IVRT was higher in Nx rats than in sham rats (36 ± 1 vs. 27 ± 1 msec in vehicle-treated and vs. 28 ± 1 msec in sitagliptin-treated sham rats, *p* < 0.05). This increase was mitigated by sitagliptin (30 ± 2 msec, *p* < 0.05 vs. Nx and non-significant vs. sham).

B-type natriuretic peptide (BNP), a biomarker of cardiac disease, was also measured. Circulating BNP levels were increased in Nx rats compared to sham rats (1.46 ± 0.18 vs. 0.64 ± 0.05 ng/mL in vehicle-treated, and vs. 0.61 ± 0.07 ng/mL, in sitagliptin-treated sham rats, *p* < 0.05), and this increase was lower in sitagliptin-treated Nx rats (0.92 ± 0.06 ng/mL) (Figure 3C). Figure 3D shows that the expression of BNP mRNA was much higher in Nx rats than in sham rats and that a treatment with sitagliptin partially prevented this increase.

### 2.4. DPPIV Inhibition Influences the Cardiac Renin-Angiotensin System (RAS) in CKD Rats

**Figure 4:**
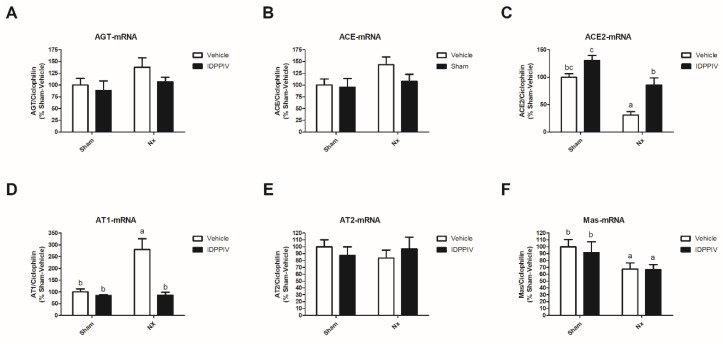
The effect of sitagliptin on the expression of cardiac renin-angiotensin system components in CKD rats. Quantitative PCR was used to determine the mRNA expression of the main components of the local renin-angiotensin system (RAS) in the heart tissue from sham rats treated with vehicle (*n* = 12) or sitagliptin (sham + IDPPIV, *n* = 9) and CKD rats treated with vehicle (Nx, *n* = 10) or sitagliptin (Nx + IDPPIV, *n* = 10). (**A**) Angiotensinogen (AGT); (**B**) angiotensin-converting enzyme (ACE); (**C**) angiotensin-converting enzyme-2 (ACE2); (**D**) angiotensin II type 1 receptor (AT1R); (**E**) angiotensin II type 2 receptor (AT2R) and (**F**) mas receptor expression (MasR). Cyclophilin was used as an internal control. The data represent the mean ± SEM. Bars with different lowercase letters are significantly different (*p* < 0.05).

**Figure 5:**
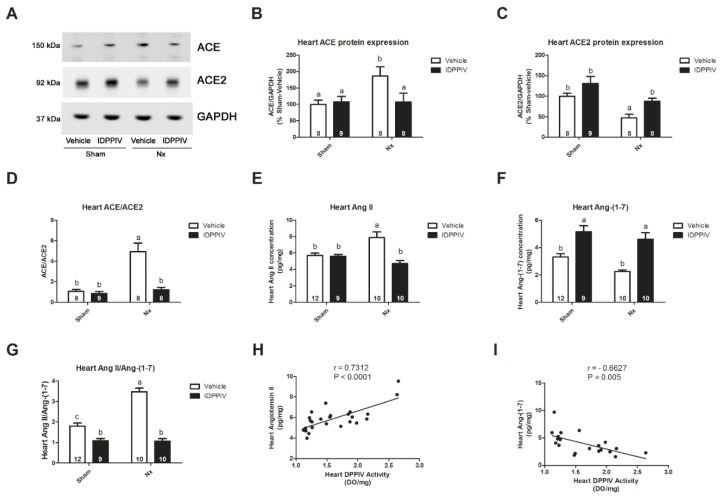
The effect of sitagliptin on the expression of ACE and ACE2 and on the concentrations of angiotensin II (Ang II) and angiotensin-(1-7) Ang-(1-7) in the heart of CKD rats. (**A**–**D**) The levels of ACE and ACE2 protein were measured in the heart tissue from sham rats and CKD rats treated with vehicle or sitagliptin (IDPPIV) by immunoblotting. (**A**) Representative immunoblot; (**B**) graphical representation of the relative levels of ACE protein in the rat. The GAPDH protein level was used as an internal control for normalization; (**C**) graphical representation of the relative levels of ACE2 protein in the rat; (**D**) the ratio of ACE to ACE2 protein expression; (**E**–**G**) the levels of Ang II and Ang-(1-7) were measured in the heart tissue from sham, sham + IDPPIV, Nx and Nx + IDPPIV rats using ELISA. The heart peptide content was normalized to protein concentration and reported as a pg/mg protein; (**E**) Ang II concentration; (**F**) Ang-(1-7) concentration; (**G**) the ratio of Ang II to Ang-(1-7) concentration. The number of rats per experimental group is indicated in the bars. The data represent the mean ± SEM. Bars with different lowercase letters are significantly different (*p* < 0.05); (**H**,**I**) correlations between the heart DPPIV activity and (H) levels of Ang II in the heart tissue; (I) levels of Ang II in the heart tissue from sham, sham + IDPPIV, Nx and Nx + IDPPIV rats. The correlation coefficients and *p* values were obtained using the Pearson correlation test, and the lines represent linear regression plotting.

The effect of sitagliptin on the components of the cardiac RAS was evaluated, and the results are shown in Figure 4 and Figure 5. A non-significant trend toward an increase in the mRNA levels of angiotensinogen (Figure 4A) and ACE (Figure 4B) was observed in the heart tissue from Nx rats compared to that from sham rats. The heart tissue from Nx rats also displayed lower levels of cardiac ACE2 mRNA (Figure 4C) and higher levels of AT1R mRNA (Figure 4D) compared to the tissue from sham rats. No differences were observed in the cardiac AT2R mRNA expression (Figure 4E). The sitagliptin treatment completely prevented the changes induced by a 5/6 nephrectomy on both the cardiac ACE2 and AT1R mRNA expression (Figure 4B,D). The mas receptor (MasR) mRNA expression was lower in the heart of CKD rats compared to sham rats (Figure 4F) and the sitagliptin treatment was not able to attenuate this reduction.

We found that a 5/6 renal ablation increased the protein abundance of ACE (Figure 5A,B) and induced a decrease in the ACE2 protein expression (Figure 5A–C) in the heart, consequently leading to a higher ratio of the cardiac *ACE* to *ACE2* expression in Nx rats (Figure 5D). The sitagliptin treatment completely abrogated the changes in the ACE and ACE2 expression as well as in the ACE to ACE2 ratio in the heart that were induced by renal ablation.

The increased ratio of the cardiac *ACE* to *ACE2* expression was accompanied by a net production of Ang II with reduced levels of Ang-(1-7) (Figure 5E–G). The sitagliptin treatment significantly reduced the levels of Ang II in the heart tissue from CKD rats (Figure 5E). Interestingly, the sitagliptin treatment enhanced the levels of Ang-(1-7) in the heart tissue of both sham and CKD rats (Figure 5E). The ratio of cardiac Ang II to Ang-(1-7) was much lower in sitagliptin-treated renal ablated rats than in Nx rats (Figure 5G). As seen in Figure 5H-I, there were significant correlations between the cardiac DPPIV activity and peptide components of the RAS. The cardiac DPPIV activity correlated positively with cardiac Ang II levels (Pearson *r* = 0.7312; *p* < 0.0001; Figure 5H) and negatively with Ang-(1-7) levels (Pearson *r* = −0.6627; *p* = 0.005; Figure 5I).

**Figure 6:**
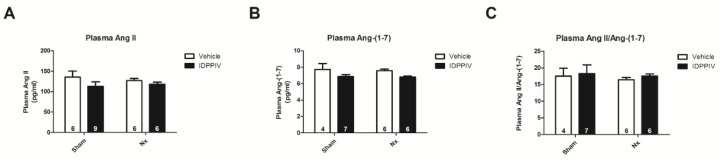
The serum concentrations of Ang II and Ang-(1-7) of sham and CKD rats are not significantly affected by sitagliptin. The levels of Ang II and angiotensin 1-7 Ang-(1-7) were measured in the serum from sham, sham + IDPPIV, Nx and Nx + IDPPIV rats using ELISA. (**A**) The serum Ang II concentration; (**B**) serum Ang-(1-7) concentration; (**C**) the ratio of Ang II to Ang-(1-7) concentration in the serum. The number of rats per experimental group is indicated in the bars. The data represent the mean ± SEM.

As shown in Figure 6, the serum levels of Ang II (Figure 6A) and that of Ang-(1-7) (Figure 6B) were similar among the four groups of rats. Additionally, no difference was observed in the ratio of serum Ang II to Ang-(1-7) (Figure 6C).

### 2.5. DPPIV Inhibition Exerts Antioxidant and Anti-Inflammatory Actions in the Heart of CKD Rats

**Figure 7:**
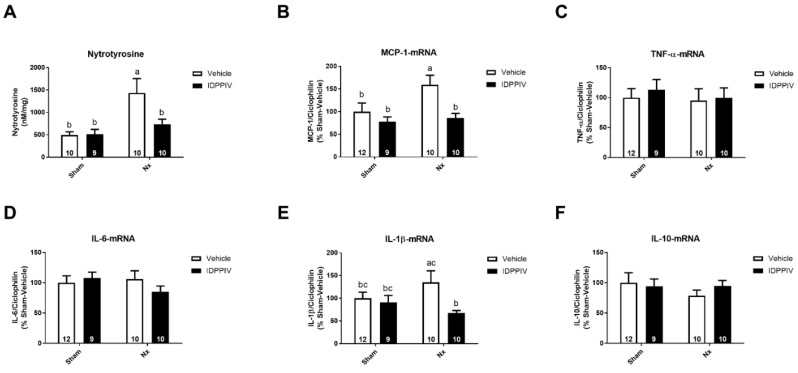
Treatment with sitagliptin exerts antioxidant and anti-inflammatory effects in the heart of CKD rats. (**A**) The levels of nitrotyrosine were measured by ELISA in the heart tissue from sham and CKD rats treated with vehicle or sitagliptin (IDPPIV); (**B**–**F**) quantitative PCR was used to determine the mRNA expression of inflammatory cytokines in the heart tissue from sham, sham + IDPPIV, Nx and Nx + IDPPIV rats. Graphical representation of the relative gene expression of (**B**) MCP-1, (**C**) TNF-α, (**D**) IL-6, (**E**) IL-1β and (**F**) IL-10. Cyclophilin was used as an internal control. The number of rats per experimental group is indicated in the bars. The data represent the mean ± SEM. Bars with different lowercase letters are significantly different (*p* < 0.05).

To investigate possible antioxidant effects of sitagliptin in the heart of rats subjected to a 5/6 renal ablation, we assessed one of the oxidative stress markers, nitrotyrosine. As shown in Figure 7A, Nx rats exhibited higher levels of nitrated proteins in the heart compared to sham rats, whereas treatment with sitagliptin significantly mitigated this increase.

The potential anti-inflammatory effects of DPPIV inhibition in the heart in our experimental model were evaluated using a quantitative analysis of the cytokine gene expression (Figure 7). Renal ablation increased the expression of MCP-1 mRNA (Figure 7B) but did not significantly alter the expression of TNF-α (Figure 7C), IL6 (Figure 7D), IL-1β (Figure 7E) and IL10 (Figure 7F) mRNA compared to sham rats. The inhibition of DPPIV by sitagliptin maintained the levels of MCP-1 mRNA that were unaltered compared to the levels in sham rats and lower than in Nx rats (Figure 7B). In addition, sitagliptin-treated rats displayed a lower expression of IL-1β mRNA than Nx rats (Figure 7E).

## 3. Discussion

The interplay between the renal and cardiac dysfunction is intricate. The progression of kidney disease and the progression of cardiac remodeling share common bidirectional pathways related to metabolic alterations, neuro-hormonal activation and the upregulation of inflammatory cascades [20,21]. The present study confirms and extends the accumulating evidence for the involvement of DPPIV in the development of cardiorenal syndromes. Our data demonstrate that the administration of the DPPIV inhibitor sitagliptin to rats subjected to a 5/6 renal ablation attenuates cardiac hypertrophy, interstitial fibrosis and diastolic dysfunction, suggesting that the DPPIV inhibitors can attenuate the progression of CVD in the setting of CKD. Moreover, our data show that the cardioprotection conferred by DPPIV inhibition in CKD rats is associated with a reduction in the cardiac levels of Ang II and an augmentation in the levels of angiotensin-(1-7), which were accompanied by pronounced local anti-hypertrophic, anti-inflammatory and antioxidant effects.

The worsening of the renal function is a common pathogenic mechanism that leads to the progression of cardiac remodeling and heart failure. The renoprotective effects of DPPIV inhibition have been consistently reported in studies in type 2 diabetic patients and in experimental models of diabetic and non-diabetic animals [1,2,3,4,5,8,22]. Indeed, gliptins are known to mitigate glomerular and tubular damage, lower proteinuria and improve sodium and water handling in a glucose-independent manner [3,5,8,22,23,24]. Herein, we found that DPPIV inhibition reduces important hallmarks of cardiovascular mortality in a 5/6 renal ablation model of CKD rats that does not display altered glycemia. DPPIV inhibition increased the glomerular filtration rate and reduced the albuminuria and elevated blood pressure. Therefore, the sitagliptin-induced delay in the onset of cardiac impairment may result, at least in part, from the partial restoration of kidney function in rats with a 5/6 renal ablation.

Previous clinical and experimental studies from our laboratory and others have demonstrated that the higher circulating DPPIV activity and expression correlates with poorer cardiovascular outcomes in the heart failure [7,8,25]. The high DPPIV activity most likely decreases the bioavailability of cardioprotective peptides, including GLP-1, BNP and SDF-1α, and the reduced biological activity of these DPPIV substrates predicts a poor CVD prognosis. Interestingly, we have noticed a remarkable increase in the cardiac DPPIV activity and expression in the heart in rats with CKD; this increase was completely abrogated by the sitagliptin treatment. On the other hand, no differences in circulating the DPPIV activity were observed between CKD and sham rats. These findings suggest that not only the systemic action but also the local inhibition of DPPIV in the cardiac cells may play a role in the cardioprotective effects of sitagliptin. In this regard, an in vitro study by Lin et al. demonstrated that sitagliptin can inhibit the increased mRNA expression of inflammatory genes, including TNF-α, IL-6, COX-2, iNOS and NF-κB, in isolated cardiomyocytes exposed to lipopolysaccharide [26]. In addition, GLP-1 and GLP-1R agonists have been consistently shown to trigger anti-fibrotic, anti-apoptotic, antioxidant and anti-inflammatory actions in cultured cardiomyocytes [27,28,29], demonstrating that GLP-1, the major DPPIV substrate, exerts direct cardioprotective effects on cardiac cells.

In line with previous studies from our laboratory [8] and others [30], we found that a gliptin not only inhibits the activity of DPPIV but also reduces the protein and mRNA expression of the peptidase in the heart of 5/6 renal ablated rats. The mechanism by which a competitive inhibitor of the DPPIV catalytic activity may also affect its expression remains to be established. Noteworthy, Kanasaki et al. [30] have observed that the reduction of the DPPIV expression in the kidney and endothelial cells of streptozotocin-induced diabetic mice by the DPPIV inhibitor linagliptin was accompanied by the upregulation of components of the microRNA (miRNA) 29 family. In addition, these authors have demonstrated that the inhibition of microRNA 29a results in the upregulation of DPPIV transcription in vitro under baseline conditions, suggesting that microRNA 29a plays a physiological role in modulating DPPIV. Ongoing studies from our laboratory are addressing the possible role of this post-transcriptional mechanism in modulating the DPPIV expression in the heart and kidney of experimental models of cardiorenal syndrome.

In the present study, cardiac remodeling was mitigated by the treatment of CKD rats with sitagliptin. In particular, DPPIV inhibition prevented cardiac fibrosis and reduced cardiac hypertrophy in this experimental model of CKD. Moreover, we used the resting echocardiography to show that DPPIV inhibition significantly mitigates the increase in the left ventricle isovolumetric relaxation time (IVRT) in 5/6 renal ablated rats. As such, we have provided unprecedented functional evidence that sitagliptin attenuates the diastolic dysfunction in an experimental model of CKD. Chaykovska et al. previously provided molecular evidence for the beneficial effects of DPPIV on the heart in CDK rats [6]. Their study showed that the DPPIV inhibitor linagliptin normalizes the mRNA levels of fibrosis markers in the heart of CKD rats. The amelioration of cardiac fibrosis by the DPPIV inhibitors in CKD rats was also demonstrated by Connelly et al. [31]. However, in contrast to our study, these authors did not observe the effects of the sitagliptin administration on cardiac hypertrophy, echocardiography parameters and renal function. These discrepancies might be related to differences in the sitagliptin dose, which was 10-fold lower in their study than in ours. Of note, clinical evidence from a subgroup analysis of the PROLOGUE study demonstrated that the addition of sitagliptin to the conventional diabetes treatment could significantly attenuate the annual exacerbation of echocardiographic parameters of the diastolic function compared to conventional treatment alone [32]. The preventive effect of sitagliptin on the diastolic dysfunction in type 2 diabetic patients observed in the PROLOGUE study was independent of blood pressure and glycemic control.

Inappropriate activation of the RAS has been implicated in a series of events that contribute to the cardiac remodeling and dysfunction. Although the systemic RAS may play a role in the remodeling process, research over the last several decades has revealed that CVD progression is primarily influenced by the local overstimulation of the RAS in the heart [33]. The data presented herein strengthens this idea, since DPPIV inhibition altered the levels of the cardiac RAS peptides Ang II and Ang-(1-7) but did not significantly change its systemic concentrations in CKD rats. Given that recent evidence suggests a vicious cross-talk between DPPIV and the tissue RAS in cardiorenal pathologies [5,17,34], we hypothesized that the inhibition of DPPIV could confer cardioprotection at least in part by counteracting the RAS overstimulation in the heart of renal ablated rats. Consistent with this hypothesis, we found that administering sitagliptin to rats subjected to a 5/6 nephrectomy prevented the majority of changes in RAS components in the heart, which included increased levels of Ang II, reduced levels of Ang-(1-7), increased expression of the AT1 receptor and reduced expression and activity of ACE2. Normalization of the cardiac ratio of Ang II to Ang-(1-7) concentrations in the heart may underlie, at least in part, the beneficial cardiac effects of sitagliptin in CKD. In line with our findings, the DPPIV inhibitor linagliptin lowered the expression of the AT1 receptor, increased the expression of the AT2 receptor and upregulated the activity of ACE2 in the heart in rats with Ang II-induced cardiac fibrosis [34]. It is important to emphasize that rather than being redundant, the combination of DPPIV inhibitors and RAS blockers may induce synergistic renoprotective and cardioprotective effects in CKD. Indeed, the DPPIV inhibitor linagliptin and the AT1 receptor blocker telmisartan had comparable efficacies to prevent CKD progression in Nx rats [4]. Additionally, the peptidomic analysis of serum and kidney samples isolated from CKD rats treated with either linagliptin or telmisartan demonstrated that the drugs have potentially overlapping renoprotective pathways, but they primarily exert their beneficial effects via different pathways [4]. Ongoing studies from our laboratory aim to dissect the major signaling pathways mediating the cross-talk between DPPIV and Ang II and between DPPIV and Ang-(1-7) in the proximal tubule cells and cardiomyocytes.

A body of experimental and clinical evidence has demonstrated that the upregulation of NHE1, the predominant Na^+^/H^+^ exchanger isoform in the heart, has pathophysiological implications for cardiac remodeling and heart failure progression [19]. In the current study, we demonstrated that the anti-fibrotic and anti-hypertrophic effects of sitagliptin in the heart in CKD rats were associated with an inhibition of NHE1 upregulation. More specifically, we found that the NHE1 expression is upregulated in the heart in CKD rats compared to sham rats and that the treatment with sitagliptin abrogated this increase. It was recently shown that cultured cardiomyocytes isolated from spontaneous hypertensive rats (SHR) exhibit hypertrophy and a higher expression and activity of NHE1 than those from normotensive Wistar Kyoto (WKY) rats. On the other hand, the NHE1 activity and expression, as well as cell size, in cardiomyocytes isolated from SHRs treated with the DPPIV inhibitor teneligliptin were restored to the levels of those from WKY rats [18]. The molecular mechanisms by which DPPIV inhibition prevents cardiac NHE1 upregulation remain to be established. Ang II is one of the major stimulators of the NHE1 activity [35]; therefore, preventing the increase in cardiac Ang II may be at least in part responsible for the effects of DPPIV inhibition on NHE1 observed in the present study and elsewhere [18]. Interestingly, DPPIV inhibition reduces the activity of the other Na^+^/H^+^ exchanger isoform, NHE3, in the proximal tubule cells of the opossum kidney (OKP) via the inhibition of a tyrosine kinase-signaling pathway [36]. This finding raises the possibility that DPPIV inhibition may modulate NHE1 by a mechanism that does not involve Ang II.

Reactive oxygen species (ROS) and inflammatory cytokines play a key role in several cardiac remodeling processes such as cardiomyocyte hypertrophy, apoptosis, contractile dysfunction, and extracellular matrix remodeling [37]. Indeed, we found in this study that cardiac hypertrophy and fibrosis in CKD rats were accompanied by a higher concentration of the oxidative stress marker nitrotyrosine and a higher expression of the proinflammatory cytokine monocyte chemoattractant protein-1 (MCP-1) in the heart compared to sham rats. These changes were blocked by the sitagliptin administration. Thus, the cardioprotective effects of sitagliptin on CKD appear to be at least in part mediated by a complete blockage of cardiac oxidative stress and inflammation. Ang II increases ROS and cytokine production; thus, similar to the NHE1 modulation, we suggest that the mechanisms underlying the antioxidant and anti-inflammatory effects of sitagliptin may be both dependent on counteracting the effects of Ang II and Ang II-independent. The Ang II-independent antioxidant and anti-inflammatory effects of DPPIV inhibition are supported by in vitro studies in which the DPPIV inhibitor gemigliptin reduced the ROS generation, reduced the expression of NADPH oxidase (*NOX4*, *p22^phox^* subunit) mRNA induced by exposing vascular smooth muscle cells to high phosphate [38] and reduced the expression of MCP-1 [39].

Cardioprotection conferred by the DPPIV inhibitors in hypertensive rats has been shown to be either dependent or independent of its effects on blood pressure. The DPPIV inhibitor linagliptin ameliorated cardiovascular injury in Dahl-salt sensitive rats with established hypertension without altering blood pressure in these animals [40]. In contrast, the attenuation of cardiac hypertrophy in spontaneously hypertensive rats by teneligliptin was accompanied by a reduction in blood pressure [18]. In this study, we observed that sitagliptin exerted an anti-hypertensive effect in rats with a 5/6 renal ablation. Therefore, we cannot rule out that part of the beneficial effects of sitagliptin on the cardiac remodeling and diastolic dysfunction found in our study may be related to the reduction of blood pressure by sitagliptin. Importantly, the data presented here show the existence of a strong positive correlation between the DPPIV activity and Ang II in the heart, while the activity of this peptidase negatively correlates with the heart levels of Ang-(1-7) (Figure 5H,I). Indeed, findings from several in vitro and ex-vivo studies demonstrate that DPPIV has direct effects on the heart [18,41,42]. Noteworthy, the DPPIV inhibitor teneligliptin prevented the pro-hypertrophic signaling induced by Ang II in isolated cardiomyocytes [18]. Additionally, a direct interaction between DPPIV and Ang II has also been shown in the kidney, where Ang II is capable of increasing the DPPIV activity in proximal tubule cells [17]. Taken together, these data suggest that the beneficial cardiac effects of sitagliptin in CKD may be due to a combination of the interaction between sitagliptin and the tissue RAS and the blood pressure lowering effect of DPPIV inhibition.

In summary, our data demonstrates that DPPIV inhibition with sitagliptin can prevent cardiac fibrosis and attenuate the cardiac hypertrophy and diastolic dysfunction in an experimental model of CKD. The possible mechanisms underlying these cardioprotective effects of sitagliptin include the counteraction of cardiac RAS, blockade of NHE1 upregulation and antioxidant, anti-inflammatory and anti-hypertensive actions. Furthermore, the beneficial effect of sitagliptin on the heart may also be due the fact that DPPIV inhibition partially prevents CKD development. DPPIV inhibition may therefore represent a novel therapeutic approach for cardiac dysfunction in CKD.

## 4. Materials and Methods

### 4.1. Animals and Surgical Procedures

All experimental procedures were approved by the Institutional Animal Care and Use Committee of the University of São Paulo Medical School (Protocol N° 003/16, 16 March 2016) and conducted in accordance with the Guide for the Care and Use of Laboratory Animals published by the United States National Institutes of Health. Male Wistar rats (2–3 months old) weighing 200–250 g were obtained from the University of São Paulo Medical School, São Paulo, SP, Brazil. Rats were randomly assigned to sham surgery or a 5/6 nephrectomy as previously described [43]. Briefly, a 5/6 renal ablation (Nx) was performed in a single-step procedure after ventral laparotomy under anesthesia with ketamine and xylazine (50 and 10 mg/kg i.p., respectively). The right kidney was removed, and two branches of the left renal artery were ligated. Sham-operated rats underwent anesthesia, ventral laparotomy and manipulation of the renal pedicles, without the removal of any renal mass. Twenty-four hours after recovering from surgery, the rats were housed in groups of four and maintained in a temperature and humidity-controlled environment with a 12-h dark-light cycle and free access to tap water and standard chow at the Heart Institute (InCor) animal facility. Rats were then randomly divided into two groups and treated for eight weeks with sitagliptin (200 mg/kg/day b.i.d.) or vehicle (water) by oral gavage. Blood samples were drawn from the aorta at the time of death for measurements of urea and creatinine concentrations and DPPIV activity. The rats were euthanized via decapitation under deep thiopental anesthesia, and their kidneys and hearts were quickly collected and weighed.

### 4.2. Blood Pressure Measurement

Six days before the end of the treatment with sitagliptin or vehicle, the blood pressure in conscious restrained rats was measured noninvasively using tail-cuff plethysmography (BP-2000 Blood Pressure Analysis System, Visitech Systems, Apex, NC, USA). Prior to these measurements, the rats were trained in the blood pressure device to become adapted to the experimental procedures.

### 4.3. Renal Function Assessment 

Five days before the end of the treatment with sitagliptin or vehicle, the rats were individually housed and placed in metabolic cages (Tecniplast, Buguggiate, VA, Italy) for four days for urine collection as previously described [44]. Food and water consumption were determined daily and subsequently normalized to body weight. Urine samples were collected during each 24-h period and used to determine urinary flow, creatinine concentration, total proteinuria and albuminuria. Creatinine clearance was used to estimate the glomerular filtration rate (GFR).

### 4.4. Urine and Blood Analysis

Fasting glucose levels were measured using the ACCU-CHECK^®^ Performa meter (Roche Diagnostics GmbH, Mannheim, Germany). The serum urea was measured by colorimetric enzymatic hydrolysis by the urease system using a Labtest kit (Labtest, Minas Gerais, Brazil). Serum and urinary creatinine concentrations were measured using a kinetic method (Labtest) and a ThermoPlate Analyzer Plus (ThermoPlate, São Paulo, Brazil). The urinary protein excretion was determined using a Sensiprot kit (Labtest). The urinary albumin concentration was determined using an ELISA kit specific for rat urine albumin (Nephrat kit; Exocell, Philadelphia, PA, USA). The experiments were carried out in accordance with the manufacturer’s instructions.

### 4.5. Echocardiography

Doppler echocardiography was performed on the last day of the treatment with sitagliptin or vehicle as previously described [8]. Images were captured from the left lateral decubitus position (45° angle). Echocardiograms were obtained using a Sonos 550 X commercial ultrasound machine (Philips Medical System, Bothell, WA, USA) with a 12–14 MHz transducer (2 cm depth, with fundamental and harmonic imaging). The systolic and diastolic functions were quantified using parasternal images of the long and short axis and using morphometry analyses of the left ventricle. Transthoracic echocardiography was performed by two investigators who were blind to the experimental groups.

### 4.6. Biometric and Morphometric Analysis

The heart was divided into two longitudinal portions for histological and molecular analysis. Its apex was separated, fixed in 10% formalin, and embedded in paraffin. Sections (5 μm) of paraffin-embedded tissue were mounted onto slides and stained with Picrosirius red to evaluate the extent of the interstitial fibrosis and with hematoxylin and eosin to evaluate the nuclear cell volume. The acquired images were quantified on five fields of each myocardium using a computerized image acquisition system (Leica Imaging Systems, Bannockburn, IL, USA). Cardiac hypertrophy was evaluated using the average nuclear volume of 50–70 myocytes, acquired in five randomized 400× magnification fields per animal, and calculated as described previously [7]. Interstitial fibrosis was measured as the percent area occupied by collagen fibers, acquired in five randomized 200× magnification fields per animal. A single examiner, blinded to the experimental groups, performed all histological measures.

### 4.7. Determination of DPPIV Activity and Abundance

The DPPIV enzymatic activity was assessed in the serum and heart homogenate by measuring the release of p-nitroaniline resulting from the hydrolysis of glycylproline *p*-nitroanilide tosylate, as previously described [23]. The heart DPPIV abundance was analyzed using immunoblotting and quantitative RT-PCR.

### 4.8. SDS-PAGE and Immunoblotting

Hearts were homogenized in a Polymix PXSR 50 E homogenizer (Kinematica, AG, Switzerland) in ice-cold phosphate buffered saline (PBS) (10 mmol/L phosphate, 140 mmol/L NaCl, pH 7.4), containing 1 μM pepstatin, 1 μM leupeptin, and 230 μM phenylmethylsulfonyl fluoride (PMSF). The total protein concentration was determined using the Lowry method [45]. Equal protein amounts of heart homogenate were run on an SDS-PAGE polyacrylamide gel (7.5%) and transferred to a polyvinylidenedifluoride (PVDF) membrane (Immobilon-P, Merck Millipore, Darmstadt, Germany) at 350 mA for 8–10 h at 4 °C. Then, the PVDF membranes were incubated with blocking solution (5% nonfat dry milk or 5% bovine serum albumin and 0.1% Tween 20 in PBS, pH 7.4) for 1 h and overnight (4 °C) with specific primary antibodies: A mouse monoclonal antibody (mAb) against DPPIV, clone 5H8 [46].

(1:1000; Santa Cruz Biotech, Santa Cruz, CA, USA); a mAb against NHE1, clone 4F9 [47] (1:1000; Santa Cruz Biotech); polyclonal antibodies against ACE (1:1000), a polyclonal antibody against ACE2 (1:1000; ab75762, Abcam, Cambridge, MA, USA); and a polyclonal antibody against GAPDH (1:5000; ab59351, Abcam). Proteins were detected using horseradish peroxidase-conjugated secondary antibodies (1:2000; Jackson ImmunoResearch, West Grove, PA, USA). The bound antibodies were detected using an enhanced chemiluminescence system (GE Healthcare, Chicago, IL, USA) according to the manufacturer’s protocols. The visualized bands were digitized using an Image Scanner (GE HealthCare) and quantified using the Scion Image Software package (Scion Corporation, Frederick, MD, USA).

### 4.9. Quantitative RT-PCR

Total RNA was isolated from the heart tissue using a Trizol reagent (Thermo Fisher Scientific, Carlsbad, CA, USA) according to the manufacturer’s instructions. The first-strand cDNA was synthesized using the Super-Script III Reverse Transcriptase according to the manufacturer’s specifications. Quantitative real-time RT-PCRs were carried out using SYBR Green PCR Master Mix-PE (Applied Biosystems, Foster City, CA, USA) on the ABI Prism 7700 Sequence Detection System (Applied Biosystems). The oligonucleotide primers used in this study are listed in Table 2. The transcript levels of three reference genes (*beta-actin, GAPDH and cyclophilin A*) were determined (Table 1). The BestKeeper software was used to identify the best suit reference gene (*cyclophilin A*) for data normalization under our experimental conditions. The relative expression was determined using the 2^−ΔΔ*C*T^ method.

### 4.10. Assessment of RAS Components

Cardiac angiotensinogen, ACE, ACE2, AT1 receptor, AT2 receptor and Mas receptor mRNAs were measured using the quantitative RT-PCR. The expression levels of ACE and ACE2 protein in the heart were determined by immunoblotting. Ang II and angiotensin-(1-7) levels in the serum and heart homogenates were measured using the competitive ELISA (Biomatik Corporation, Cambridge, Ontario, MA, Canada) according to the manufacturers’ instructions, including the sample collection and storage. The serum was stored at –80 °C until assay. Heart homogenates were obtained as described above and stored at –80 °C. The heart peptide content was normalized to a protein concentration and reported as a pg/mg protein.

### 4.11. Heart Nitrotyrosine Levels

The nitrotyrosine levels (3-nitrotyrosine) were measured in the heart homogenate using an ELISA kit according to the manufacturer’s instructions (Hycult Biotech, Plymouth Meeting, PA, USA).

### 4.12. Statistical Analyses

The data are shown as the mean ± standard error of the mean (SEM). Comparisons among the means were assessed using the two-way analysis of variance (ANOVA) followed by the Bonferroni post hoc test. A *p* value < 0.05 was considered significant when considering the main effect of the sitagliptin treatment, the main effect of a 5/6 renal ablation, the interaction between the sitagliptin treatment and 5/6 renal ablation, and the differences among groups. The relationships between the heart DPPIV activity and the cardiac concentrations of Ang II and Ang-(1-7) were assessed by the Pearson correlation test. All statistical analyses were performed using the GraphPad Prism 5 software (GraphPad Software, La Jolla, CA, USA).

## Figures and Tables

**Table 1:** Body weight, glycemia, blood pressure and renal function parameters of sham rats, rats subjected to a 5/6 nephrectomy (Nx) and treated with vehicle (Nx) and rats subjected to a 5/6 nephrectomy and treated with the dipeptidyl peptidase IV DPPIV inhibitor sitagliptin (Nx + iDPPIV).

Physiological Parameter	Sham	Sham + IDPPIV	Nx	Nx + IDPPIV
Initial body weight, g	193 ± 4 (16)	192 ± 6 (10)	198 ± 4 (16)	202 ± 3 (16)
Final body weight, g	323 ± 7 (9)	331 ± 15 (7)	311 ± 12 (10)	337 ± 7 (10)
Body weight gain, %	67 ± 3 (9)	73 ± 5 (7)	58 ± 6 (10)	67 ± 3 (10)
Blood Glucose, mg/dL	80 ± 3 (9)	83 ± 2 (8)	79 ± 1 (10)	81 ± 2 (10)
Blood Creatinine, mg/dL	0.45 ± 0.03 ^c^ (10)	0.40 ± 0.03 ^c^ (7)	0.99 ± 0.05 ^a^ (11)	0.72 ± 0.03 ^b^ (12)
GFR, mL/min/kg	8.2 ± 0.4 ^c^ (10)	7.5 ± 0,9 ^c^ (7)	2.2 ± 0.4 ^a^ (11)	4.6 ± 0.3 ^b^ (12)
Blood Urea, mg/dL	41 ± 2 ^c^ (10)	37 ± 2 ^c^ (7)	94 ± 4 ^a^ (11)	70 ± 4 ^b^ (12)
Protein excretion, mg/24 h/kg	60 ± 7 ^c^ (10)	61 ± 5 ^c^ (7)	602 ± 86 ^a^ (11)	294 ± 43 ^b^ (12)
Albumin excretion, mg/24 h/kg	9 ± 3 ^c^ (8)	6 ± 3 ^c^ (7)	162 ± 25 ^a^ (10)	97 ± 11^b^ (10)
Albumin/creatinine	0.18 ± 0.05 ^c^ (8)	0.13 ± 0.07 ^c^ (7)	6.42 ± 0.73 ^a^ (10)	2.91 ± 0.52 ^b^ (10)
Tail cuff BP, mmHg	124 ± 5 ^c^ (11)	126 ± 5 ^c^ (9)	184 ± 5 ^a^ (12)	166 ± 5 ^b^ (11)
Serum DPPIV activity, OD	0.399 ± 0.017 ^b^ (16)	0.096 ± 0.006 ^a^ (10)	0.419± 0.012 ^b^ (16)	0.091 ± 0.009 ^a^ (16)

Values are means ± SEM. Number of animals per group is indicated in parenthesis. GFR = Glomerular filtration rate, BP = Blood Pressure, OD = Optical Density. Bars with different lowercase letters are significantly different (*p* < 0.05).

**Table 2:** Sequence of oligonucleotides used in this study.

*Gene*	Sense (S)/Antisense (AS)	*Sequence*
*ACE*	S	GACCAAAAGCTGCGAAGGAT
	AS	TTGTTGGGGAAGCAGACCTT
*ACE-2*	S	TGTGGGGTAGGTTTTGGACA
	AS	GGAAGGCCAACAGAAACGAA
*ACT-β*	S	CTGTGACATCCGTAAGACC
	AS	GCCACCAATCCACACAGA
*AGT*	S	TGGATAAAGAACCCGCCTCC
	AS	TTGAGAACCTCTCCCACTCG
*AGT R-1α*	S	TCTGCCACATTCCCTGAGTTA
	AS	CTTGGGGCAGTCATCTTGGA
*AGT R-1β*	S	AGTGACAGAGACCAGACCAGA
	AS	TTGGGGCAGTCATCTTGGATT
*AGT R-2*	S	TTTGCCATCCTCCTGGGATT
	AS	GCCTTGGAGCCAAGTAATGG
*BNP*	S	GATTCTGCTCCTGCTTTTCC
	AS	TCTTTTGTAGGGCCTTGGTC
*CYCLO*	S	AATGCTGGACCAAACACAAA
	AS	CCTTCTTTCACCTTCCCAAA
*GAPDH*	S	ATGGTGAAGGTCGGTGTG
	AS	GAACTTGCCGTGGGTAGAG
*IL-1β*	S	CCTGTGTGATGAAAGACGGC
	AS	TATGTCCCGACCATTGCTGT
*IL-6*	S	CTGGTCTTCTGGAGTTCCGT
	AS	GCCACTCCTTCTGTGACTCT
*IL-10*	S	TGGGAGAGAAGCTGAAGACC
	AS	AGATGCCGGGTGGTTCAAT
*MCP-1*	S	TGCCCACTCACCTGCTGCT
	AS	TGGGGTCAGCACAGATCTCTCTCT
*MAS1*	S	GACCAGTCATCCTGCCAGA
	AS	CATGAGGAGTTCTTGTGCTGG
*TNF-α*	S	ATCGGTCCCAACAAGGAGG
	AS	GATAAGGTACAGCCCATCTGC

ACE—Angiotensin converting enzyme; AT-1—Angotensin II receptor type 1; AT-2—Angotensin II receptor type 2; ACT—Actin; AGT—Angiotensinogen; AGT R—Angiotensinogen receptor; BNP—Brain natriuretic peptide; CYCLO—Cyclophilin; GAPDH—Glyceraldehyde 3-phosphate dehydrogenase; IL—Interleukin; MCP—Monocyte chemoattractant protein; MAS—Mas receptor; TNF—Tumor necrosis factor.
